# Evaluating the physical-mechanical properties of flowable fiber-reinforced and bulk-fill Giomer composites: a comparative study of advanced technologies

**DOI:** 10.3389/fdmed.2025.1634533

**Published:** 2025-10-13

**Authors:** Alyssa Teixeira Obeid, Arnaldo José Capellán López, Letícia Vendrametto Forcin, Nair Cristina Margarido Brondino, Rafael Francisco Lia Mondelli, Sofia Furlanete Raymundo, Abdulaziz Alhotan, Nick Silikas, Marilia Mattar de Amoêdo Campos Velo

**Affiliations:** ^1^ Department of Operative Dentistry, Endodontics, and Dental Materials, Bauru School of Dentistry, University of São Paulo, Bauru, Brazil; ^2^ Department of Mathematics, School of Sciences, São Paulo State University (UNESP), Bauru, Brazil; ^3^ Department of Restorative Dentistry, School of Dentistry, São Paulo State University (UNESP), Araraquara, Brazil; ^4^ Department of Dental Health, College of Applied Medical Sciences, King Saud University, Riyadh, Saudi Arabia; ^5^ Division of Dentistry, Faculty of Biology, Medicine and Health, The University of Manchester, Manchester, United Kingdom

**Keywords:** bulk-fill, fiber-reinforced composites, Giomer technology, mechanical properties, shrinkage stress

## Abstract

The mechanical performance and polymerization shrinkage stress of resin composites play a critical role in maintaining marginal integrity over time. This *in vitro* study compared the properties of fiber-reinforced flowable resin (FRC) and S-PRG bulk-fill flowable resin composites. Three materials were tested: EVER X (FRC), S-PRG (Giomer tecnology), and SDR (conventional bulk-fill). Analyzed variables included surface hardness (SH), depth of cure, flexural strength (FS), modulus of elasticity (E), polymerization shrinkage stress, and degree of conversion (DC). Scanning electron microscopy (SEM) was employed to assess composite morphology. Samples were prepared for SH, depth of cure, and DC measurements. A three-point bending test evaluated FS and E, while shrinkage stress was measured between parallel steel plates. A strong positive correlation was found between FS and E. S-PRG exhibited the highest shrinkage stress, followed by EVER X and SDR. SEM images of fractured EVER X samples showed fiber-pulling effects, indicating internal reinforcement. EVER X demonstrated superior mechanical properties—including high flexural strength, surface hardness, degree of conversion, and elastic modulus—making it a promising option for load-bearing restorations. S-PRG, with its excellent depth of cure, may be more suitable for situations requiring deeper light penetration.

## 1 Introduction

Flowable resin composites have undergone significant advancements since their initial application in Class V cavity restorations. Over time, improvements in their formulations and the incorporation of new technologies have broadened their use across various dental applications, including the treatment of small carious lesions, crown cementation, pit and fissure sealants, cavity liners, and rebuilding contact areas in resin composite restorations (1, 2). Specifically designed to streamline and expedite the restorative process, bulk-fill flowable resin composites allow for incremental placements of 4–5 mm, reducing volumetric shrinkage during polymerization (3). While bulk-fill flowable composites enhance efficiency and simplify dental restorations (4), concerns regarding their mechanical properties remain (5, 6).

Fiber-reinforced flowable resin composites (FRCs) have been introduced as suitable materials for high-stress areas in dental restorations. With incremental depths of up to 4 mm, FRCs provide the strength and durability required for bulk filling as a dentin replacement, supporting final restorations and mimicking the toughness and resilience of natural dentin (7–9). Their strong adhesive properties also help reduce clinical issues such as microleakage, secondary caries, and restoration dislodgement (10). Despite these advantages, there is a significant gap in the literature regarding the polymerization shrinkage behavior of FRCs in comparison to other resin composites. Since polymerization shrinkage directly affects marginal adaptation, this knowledge is essential for understanding their long-term clinical performance.

Marginal infiltration remains the primary cause of restoration failure, responsible for over 50% of replacement cases. In response, advanced hybrid composites have been developed to improve cavity wall adaptation and enhance marginal sealing—critical factors in extending restoration longevity (11, 12). A notable advancement in this field is Giomer technology (*S-PRG;* Surface Pre-Reacted Glass-ionomer), which combines the anti-caries and self-adhesive benefits of glass ionomers with the mechanical strength and aesthetic appeal of resin composites. More recently, Giomer has been incorporated into bulk-fill flowable materials, further expanding its potential applications in restorative dentistry (13).

Since over 60% of restorations are replaced due to failure—often resulting in significant damage and expense—comparative studies on the physical and mechanical properties of these advanced materials are both necessary and limited. To optimize restorative outcomes, professionals should consider factors such as the long-term durability of the materials used (14).

This *in vitro* study aimed to compare the physical and mechanical properties of FRC and Giomer-based bulk-fill flowable resin composites, using standardized methodologies to minimize variability. The surface hardness (SH), depth of cure, flexural strength (FS), modulus of elasticity (E), polymerization shrinkage stress, degree of conversion (DC), and surface morphology were evaluated. The null hypothesis was that there are no significant differences in the physical-mechanical properties between FRC and Giomer-based bulk-fill flowable resin composites.

## 2 Materials and methods

### 2.1 Experimental design

This *in vitro* study evaluated three commercially available flowable resin composites, each representing a distinct type of composite and technology: (1) fiber-reinforced flowable resin composite (EVERX), (2) bulk-fill flowable resin composite with Giomer technology (S-PRG), and (3) conventional bulk-fill flowable resin composite (SDR). Table 1 provides detailed information on these restorative materials. The analyzed response variables included SH, depth of cure, FS, E, polymerization shrinkage stress, and DC. The morphology of the composites was also assessed using a scanning electron microscope (SEM).

**Table 1:** Specifications of the materials used.

Resin composites	Color	Manufacturer	Composition	Viscosity	Lot number
EverX Flow Bulk	Bulk Shade	GC, Toquio, Japan	UDMA, Barium Glass: 42%–52%	Flow bulk	2107201
			Silicon Dioxide: Trace, Bis-MEPP: 15%–25%		
			TEGDMA: 1%–10%		
			UDMA: 1%–10%		
			and E-glass fibers 25%		
Beautifill Bulk-flow (S-PRG)	A3	Shofu, Kyoto, Japão	Nano-Hybrid Composite with Fluoride Release and Recharge	Flow bulk	062384
SDR Surefill	Universal Shade	Dentisply, Sirona, Waltham, MA, EUA	(EBPADMA), (TEGDMA), (CQ), Barium boron fluoride aluminum silicate glass, strontium aluminum fluoride silicate glass.	Flow bulk	00099347

### 2.2 Microhardness assessment (KHN)

Disc-shaped samples (4 × 2 mm²) were prepared (*n* = 6) by placing the material into stainless-steel moulds, covering it with a polyester strip, and light-curing through the strip for 40 s (Valo Grand, Ultradent). Three indentations were made on the top and bottom surfaces of each sample along a middle line, spaced 100 μm apart (Knoop diamond, 50 g, 15 s dwell time), using digital microhardness equipment (Micromet II, Buehler, USA). The mean of the three readings was obtained.

### 2.3 Bottom-to-top hardness ratio from Knoop microhardness (SH)

The average of the three surface readings (taken from both the top and bottom) was calculated, and the hardness ratio between the bottom and top surfaces was determined (15, 16).

### 2.4 Degree of conversion (DC)

The DC of the samples (*n* = 6) was measured using a Fourier transform infrared spectroscope (FTIR, Shimadzu Corporation, Model IR Prestige 21, Kyoto, Japan) equipped with an attenuated total reflectance (ATR-Smart Miracle™) accessory. Measurements were conducted in a naturally lit environment with approximately 45% air humidity and a controlled temperature of 20 °C. For the unpolymerized spectrum, about 3 μl of the flowable resin was placed on the ATR crystal to measure absorption bands. The sample was then light-cured using the Valo device, with an irradiance of 1,200 mW/cm² for the time recommended by the manufacturer of each resin.

The DC percentage was then calculated for each sample, using the following formula:

$$\text{\%}DC=\frac{[1-peak\;height\;after\;curing]\;\times 100}{Peak\;height\;before\;curing}$$

### 2.5 Flexural strength (FS) and modulus of elasticity (E)

Ten bars from each group (8 × 2 × 2 mm³) were fabricated using a bipartite steel matrix, following a modified ISO 4049 standard (17–19). Each type of resin composite was placed in a single increment and light-cured for 20 s using the Valo device at 1,200 mW/cm². The samples (*n* = 10) were stored in a dry oven at 37°C for 24 h prior to the FS test. FS was determined using a three-point bending test on a universal testing machine (Instron, Barueri, SP, Brazil) equipped with a 500 N load cell at a constant test speed of 0.5 mm/min. FS values were calculated according to the following formula:

$$FS=3Fl/2bh^{2}$$

Where, *F* is the maximum load exerted [N], I is the distance between the supports [6 mm], b is the width and h is the height of the specimen [mm].

To calculate the modulus of elasticity, the following formula was used:

$$E=F1l^{3}/4bh^{3}d$$

F1 is the load at a point in the straight line portion of the load/displacement curve [N], and d is the deflection at load F1 [mm].

### 2.6 Polymerization shrinkage stress

To measure shrinkage stress, the respective resin composites were placed in a single increment between two rectangular steel plates (6 × 2 mm²), arranged parallel to each other, maintaining a constant resin volume of 12 mm³ (16, 17, 20). These plates were connected to a 50 kg/F load cell in a universal testing machine, and the resin was light-cured for 20 s using the Valo device at 1,200 mW/cm². To standardise light-curing process, the light source was positioned 1 mm from the sample to ensure proper light transmission through the entire resin increment (16). Throughout the polymerization process, the contraction forces (*N*) generated by the composites were recorded from the initiation of light-curing up to 300 s (16, 21, 22). The data were captured by UTM software and presented as a force (*N*) vs. time (s) graph. Shrinkage stress (MPa) was calculated by dividing the contraction force (*N*) at 300 s by the cross-sectional area (mm²) of the metallic plates. All measurements were conducted by a single operator at room temperature (25 °C).

### 2.7 Scanning electron microscopy (SEM)

After flexural testing, samples (*n* = 3) were prepared for microscopy. They were first positioned on a metallic base to enable ionisation for surface analysis. The samples were then examined under a microscope (JEOL model T 220 A), capturing images of the increments at 50x and 500x magnifications. These images were selected to evaluate the surface of each resin after fracture.

### 2.8 Statistical analysis

All analyses were conducted considering a significance level of *α* = 5%. All statistical analyses, except for the DC, were performed using R software (R Core Team, 2024). The analysis of the DC was conducted using R software, version 4.0.0 (R Core Team, 2019). To ensure blinding of the statistician, numerical codes (1–3) were assigned to the groups prior to data disclosure (23).

For the FS analysis, a generalized linear model with an inverse Gaussian distribution for the response variable and a log link function was employed. Deviance analysis was conducted to assess the effect of group, and pairwise comparisons were performed using Tukey's *post hoc* tests. Given the violation of the homoscedasticity assumption, robust estimators of variance were adopted in the inferential procedures.

Regarding the E, group comparisons were performed using one-way ANOVA, followed by Tukey's tests for pairwise comparisons.

For bottom SH, a generalized linear model with a Gamma distribution and a log link function was used. Deviance analysis identified significant group effects, and *post hoc* comparisons were carried out using Tukey's tests. For top SH, one-way ANOVA was used to compare groups. Due to the violation of the homoscedasticity assumption, robust variance estimators were applied.

To model the depth of cure, a weighted least squares regression was employed due to the observed violation of the homogeneity of variance assumption across groups. In all models, the normality of residuals was assessed through Q-Q plots with simulated envelopes, while residuals vs. predicted values plots were examined to evaluate homoscedasticity. For DC, after confirming the normality and homogeneity of the data, a nonparametric test (Kruskal–Wallis) was performed.

## 3 Results

### 3.1 Top and bottom hardness and depth of cure

**Figure 1:**
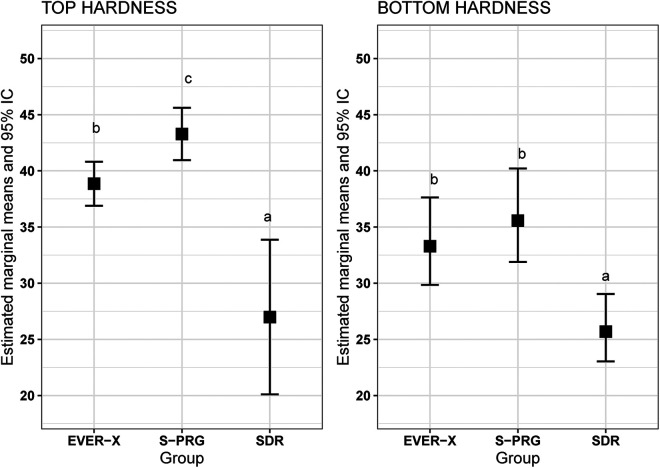
Estimated marginal means and 95% CI for hardness at the **(A)** top and **(B)** base of the samples.

For top SH, the EVER X and S-PRG exhibited the highest mean hardness values, while the SDR group demonstrated the greatest variance. Since the assumption of homoscedasticity was rejected, robust estimators were used to construct the confidence intervals. ANOVA indicated statistically significant differences among the groups (*p* < 0.0001). Therefore, pairwise comparisons were performed using Tukey's method. Significant differences were found between the EVER X and S-PRG groups (*p* = 0.005), between EVER X and SDR (*p* = 0.0007), and between S-PRG and SDR (*p* < 0.0001). The estimated marginal means and their 95% confidence intervals are presented in Figure 1.

The deviance analysis indicated significant differences among the groups (*p* = 0.0002). Therefore, pairwise comparisons were performed using Tukey's method. No significant difference was found between the EVER X and S-PRG groups (*p* = 0.53). However, significant differences were observed between EVER X and SDR (*p* = 0.002), and between S-PRG and SDR (*p* = 0.003) (Figure 1).

**Figure 2:**
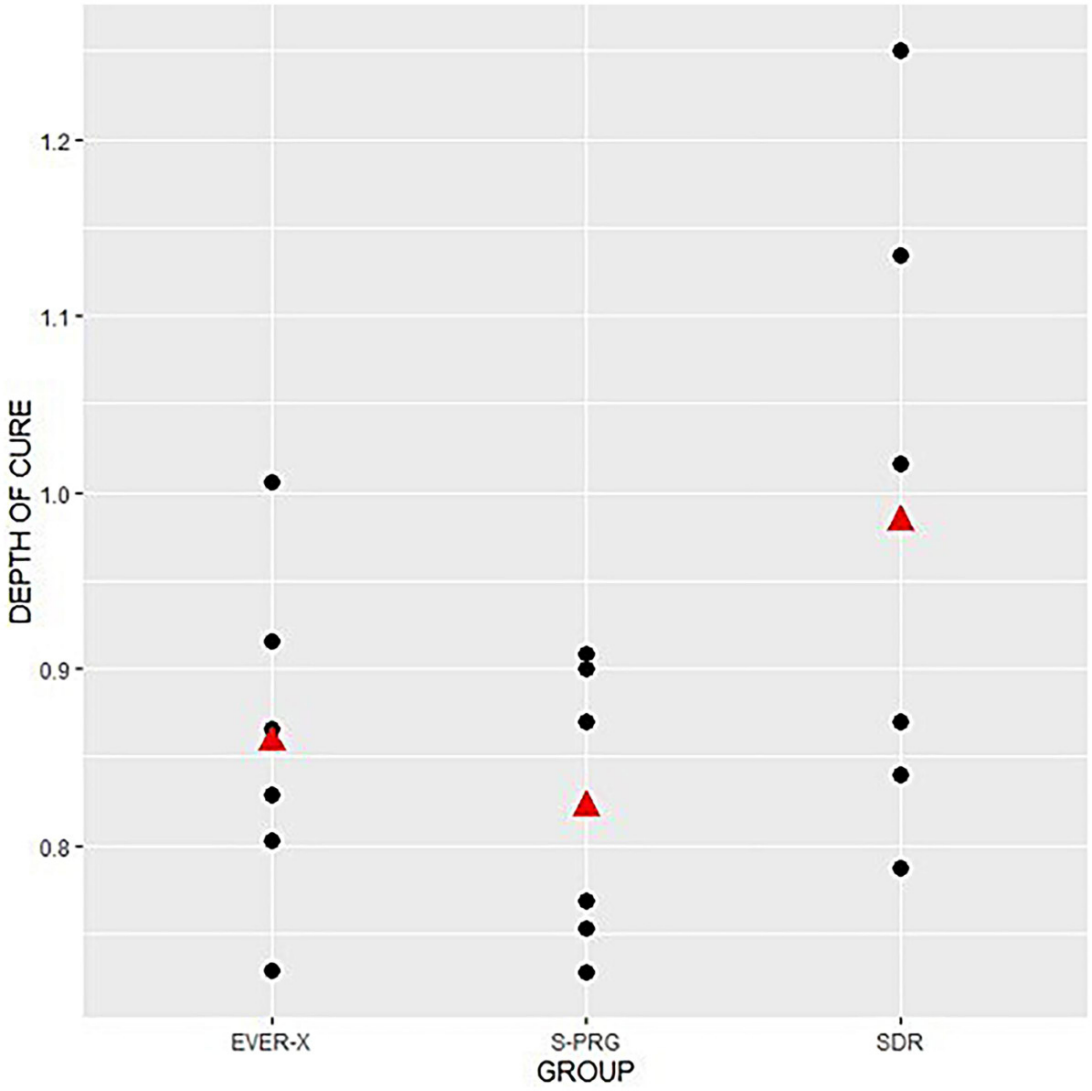
Individual values graph for depth of cure.

The individual value plot shown in Figure 2 illustrates the depth of cure profile for the three studied groups. As observed, the means and variances for the EVER X and S-PRG groups were similar, whereas both the mean and variance for the SDR group were higher. Due to the presence of heteroscedasticity, a weighted regression was performed, assigning greater weights to observations with lower variances. The results did not indicate significant differences in cure depth among the three groups (*p* = 0.17).

### 3.2 Flexural strength, modulus of elasticity and degree of conversion

The deviance analysis indicated significant differences among the groups (*p* = 0.006), suggesting that the materials performed differently in the flexural strength test. According to Tukey's test, no significant difference was found between the S-PRG and SDR groups (*p* = 0.78). The comparison between the EVER X and SDR groups also did not show a statistically significant difference at the 5% level (*p* = 0.059). However, a significant difference was observed between the EVER X and S-PRG groups (*p* = 0.02).

**Figure 3:**
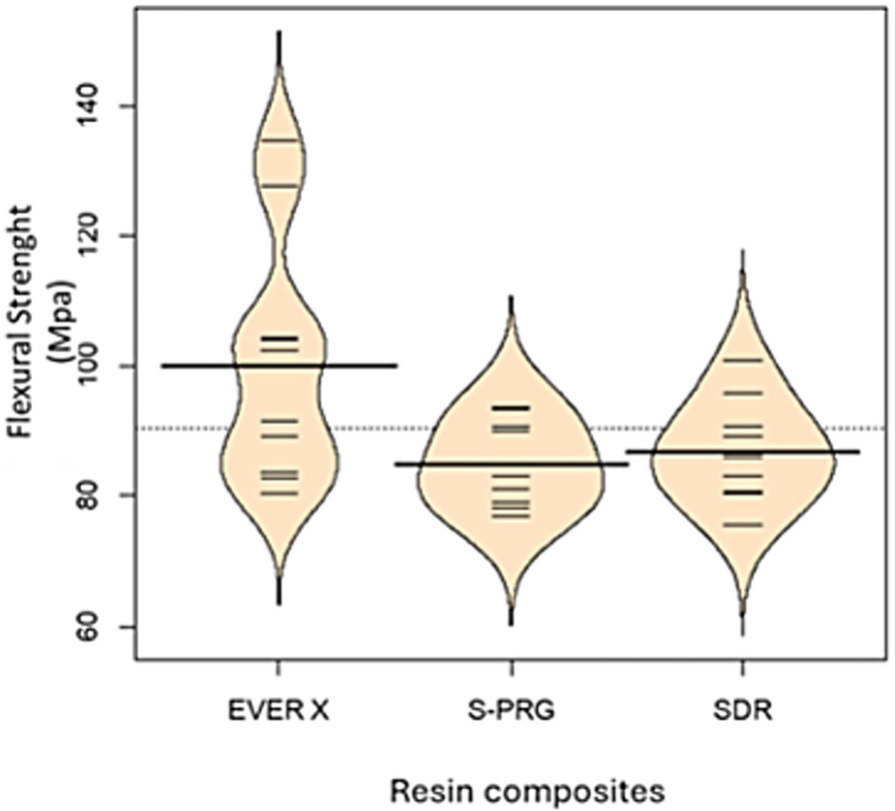
Bleanplot for three-point bending test.

The beanplot in Figure 3 illustrates the distribution of test results for each group. The larger horizontal lines represent group means, while the smaller lines correspond to individual observations. The outline suggests the distribution of values, with the S-PRG and SDR groups showing more symmetrical distributions concentrated around the mean. In contrast, the EVER X group exhibits an asymmetrical distribution with greater dispersion around the mean. EVER X showed higher FS values (*p* < 0.05) (Figure 3).

For modulus of elasticity, the test results showed no significant difference between the EVER X and S-PRG groups (*p* = 0.25). However, significant differences were found in the comparisons between the EVER X and SDR groups (*p* < 0.0001) and between the S-PRG and SDR groups (*p* = 0.0007). These pairwise comparisons are summarised in Table 2. The results showed a significant difference in the DC between EVER X and S-PRG (*p* < 0.05) (Table 2).

**Table 2:** Mean ± standard deviation (SD) values of FS and E of the studied groups.

Resin composites studied	Degree of conversion (%, *n* = 6)	Modulus of elasticity (*n* = 10, Gpa)
EVER X	60.20 (59.2–62.1)^ab^	5.83 ± 0.4^b^
S-PRG	36.07 (33.3–39.1)^c^	5.52 ± 0.5^a^
SDR	53.45 (51.8–45.9)^ac^	4.72 ± 0.35^ab^

* Distinct lowercase letters between lines indicate statistically significant differences between groups.

**Figure 4:**
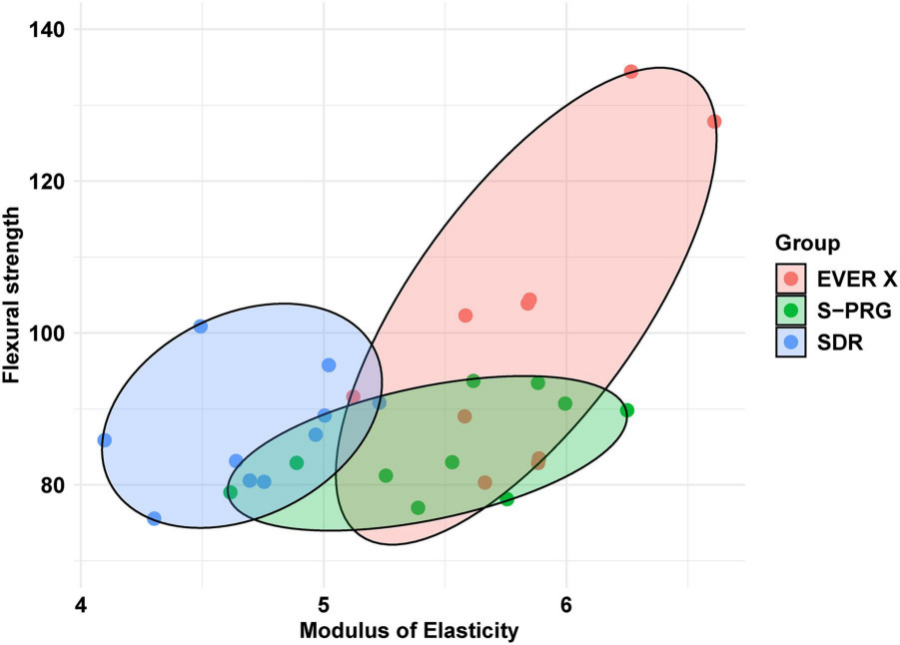
Combined behavior of the flexural test and modulus of elasticity.

The results indicate a strong positive correlation between higher flexural strength values and higher modulus of elasticity values for the SDR and EVER X groups, as evidenced by the steep inclination of the ellipses in Figure 4. A similar positive correlation was observed for the S-PRG resin, although it was less pronounced, as indicated by the reduced inclination of the ellipse.

### 3.3 Polymerization shrinkage stress and SEM images

**Figure 5:**
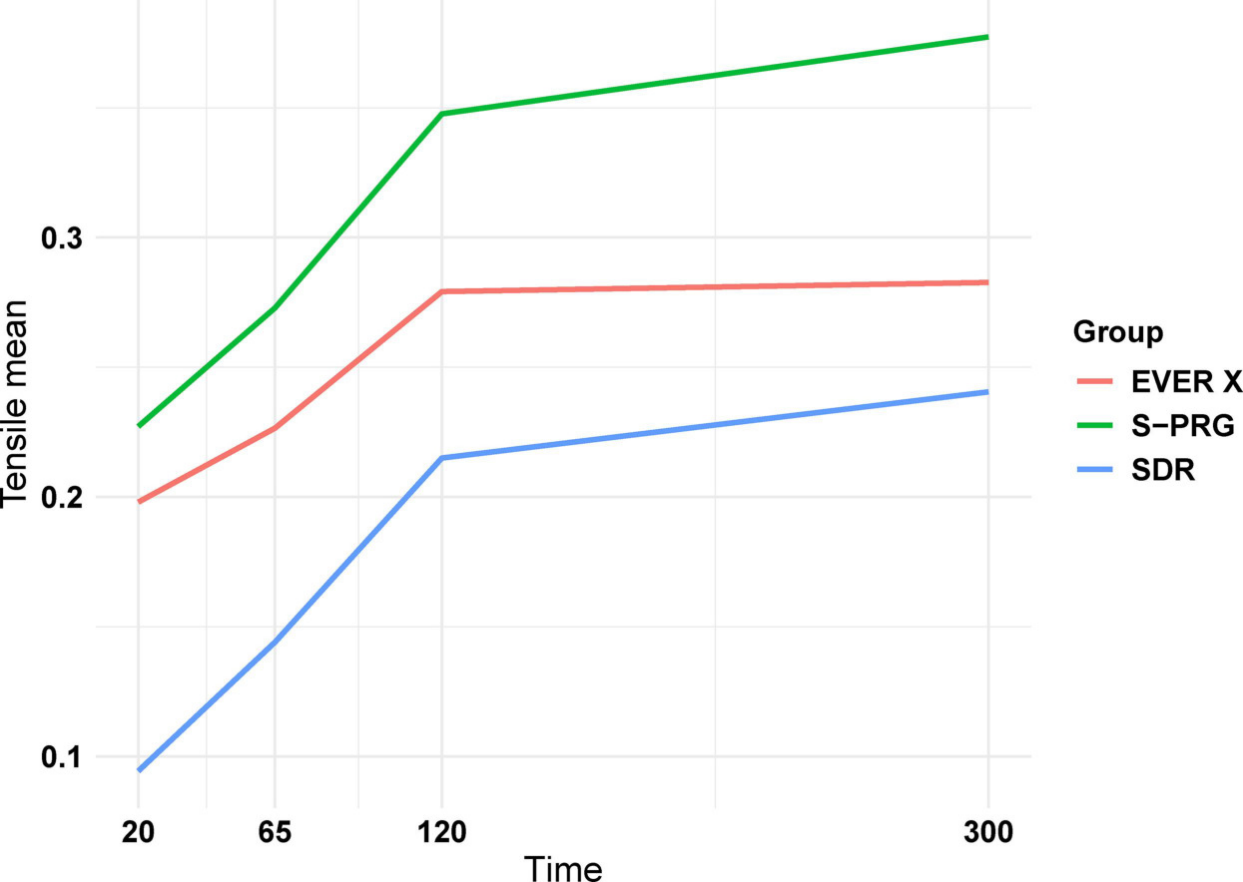
Shrinkage stress as a function of time.

The graph in Figure 5 illustrates the behaviour of group averages based on measurements taken over time.

As shown, there was a pronounced increase in tension during the first 120 s. Between 120 and 300 s, the mean exhibited reduced variability over time. The graph indicates that the mean shrinkage stress for the S-PRG composite is higher than that for the EVER X resin, which, in turn, is higher than that for the SDR group. The curvature observed suggests that tension behavior over time is nonlinear.

**Figure 6:**
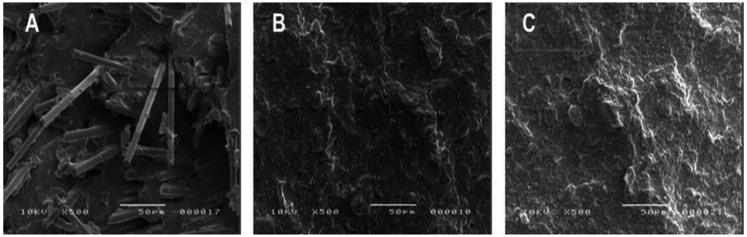
Images of fractured aesthetic s of **(A)** EVER X, **(B)** SDR and, **(C)** S-PRG resin composite at 500x.

SEM images (Figure 6) revealed irregular surfaces of the resin composites after fracture. The EVER X resin remained embedded within the matrix after fracture (Figure 6C), exhibiting a “pulling out” effect from the matrix. This fracture surface pattern differed from those of the other resins.

## 4 Discussion

Despite advancements in inorganic filler systems and the development of microhybrid and nanohybrid resins, resin composites continue to face issues with microleakage and polymerization shrinkage, which directly contribute to higher fracture rates and an increased risk of recurrent caries (24). Consequently, researchers have focused on developing resin composites with improved physical and mechanical properties, as well as bioactive potential, which could be particularly beneficial for patients at higher risk of developing caries.

Resin composites with advanced technologies have emerged as solutions for large posterior restorations due to their enhanced mechanical properties and simplified application processes. In this context, FRCs, like EVER X, have gained significant attention in restorative dentistry. This resin, reinforced with short glass fibers, enhances fracture toughness, making it particularly suitable for high-stress areas, such as posterior fillings, core build-ups, and large cavities. The fiber content provides superior resistance to crack propagation due to the load-bearing capabilities of the embedded fibers, which distribute stress and prevent catastrophic failure under occlusal forces. SEM images (Figure 6A) show a distinct surface morphology in EVER X, with fibers pulling out, indicating interlocking resistance within the matrix. This interlocking requires higher fracture energy, contributing to improved durability (17, 23, 25). EVER X also demonstrated significantly higher SH at the top (*p* < 0.05, Figure 1A), likely due to a well-distributed filler content. However, previous studies have shown that EVER X may exhibit reduced durability under prolonged water exposure compared to non-fiber-reinforced materials (26).

Like fiber-reinforced bulk-fill resins, S-PRG bulk-fill composites allow for bulk placement in deep cavities, reducing chair time and improving clinical efficiency. This capability makes them suitable for large restorations by facilitating faster application without compromising the restoration's integrity or longevity. However, S-PRG-based resins differ in composition, properties, and clinical performance. Some studies have indicated that bulk-fill S-PRG materials may exhibit reduced FS and compressive strength, both of which are critical for clinical performance (27). The DC for bulk-fill S-PRG materials tends to be lower due to the high filler content, which affects light penetration during curing (28). In the current study, EVER X exhibited the highest DC (*p* < 0.05, Table 2), which can be attributed to the large surface area of the nanofibers, promoting increased intermolecular hydrogen bonding between the nanofibers and resin matrix. The bulk-fill composite SDR also showed high DC values, primarily due to fillers with closely matched refractive indices, which minimize light scattering and allow deeper light penetration during curing (29). High DC values are essential for achieving the mechanical properties required for long-term restorations and for reducing polymerization shrinkage stress, a common issue in deeper cavities (30, 31).

In deeper restoration layers, polymerization levels are typically lower (32). Therefore, depth of cure was evaluated, revealing similar results across all groups (Figure 2, *p* > 0.05). The translucency of SDR and the fiber content in EVER X allow effective polymerization in deeper layers. Likewise, the closely matched refractive indices of the fillers in the S-PRG composite reduce light scattering, allowing for deeper curing. Literature on polymerization shrinkage in flowable bulk-fill giomers and FRC resins is limited; in this study, EVER X exhibited the lowest polymerization shrinkage stress over time (Figure 5) in comparison to S-PRG material, which may help minimize microleakage and marginal breakdown. Generally, restorative materials with fiber reinforcement and higher aspect ratios tend to interlock within the cavity, effectively reducing polymerization shrinkage stress, as corroborated by previous studies (33–35).

Elastic modulus is also an important factor in preventing microleakage and restoration dislodgement as it relates to material stiffness. A high modulus of elasticity is desirable to resist deformation under masticatory stresses. Resilience is particularly important for flowable materials layered beneath a conventional composite for load-bearing applications. Figure 4 demonstrates a strong positive correlation between FS and modulus of elasticity for SDR and EVER X. Previous research has shown that fiber-reinforced composite resins and fiber-reinforced posts can effectively restore endodontically treated teeth, including those with extensive structural damage, by enhancing their fracture resistance (36–38). Fiber reinforcement in EVER X significantly reduces shrinkage stress and provides a higher modulus of elasticity, making it more suitable for restorations where minimizing stress is critical. Conversely, SDR displayed moderate DC and a lower modulus of elasticity (4.72 GPa), suggesting reduced stress resistance. While suitable for lower-stress applications, its lower modulus may affect margin integrity under occlusal forces, making it less ideal for high-stress restorations.

In summary, EVER X's higher modulus of elasticity and lower polymerization shrinkage may make it advantageous for maintaining marginal integrity, especially in load-bearing areas. Clinical trials will be crucial for evaluating the long-term durability of these new technologies, as they enable a comparative assessment of materials under oral conditions.

In a previous clinical study, it was showed that using a flowable fiber-reinforced composite without any proximal surface coverage in Class II restorations resulted in general, in satisfactory clinical outcomes in parameters such as anatomical contour, retention, surface texture, and marginal integrity, over an 18-month follow-up period (39). Additionally, a systematic review of *in vitro* studies has demonstrated that short fiber-reinforced composites can effectively reinforce structurally compromised teeth (9). In studies comparing restorations using FRCs with and without a particulate-filled composite as a covering, the groups restored with FRCs often demonstrated superior mechanical behavior (9). These findings indicate that, when applied under well-established clinical protocols, short fiber-reinforced composites may represent a promising option for single-layer restorative procedures.

## 5 Conclusion

The results suggest that EVER X, due to its high flexural strength, satisfactory surface hardness, degree of conversion, elastic modulus, and low polymerization shrinkage stress, is well-suited for load-bearing restorations and cases where minimizing stress is key to preserving marginal integrity. S-PRG showed good depth of cure, making it useful in situations requiring deeper light penetration. However, its higher shrinkage stress and lower mechanical strength may limit its use in high-stress restorations, despite its anti-caries properties. SDR demonstrated a moderate degree of conversion and a low elastic modulus, suggesting that it may be more suitable for low-stress areas but less appropriate for restorations subjected to high occlusal forces.

## Data Availability

The raw data supporting the conclusions of this article will be made available by the authors, without undue reservation.
